# Advancements in Cataract Detection: The Systematic Development of LeNet-Convolutional Neural Network Models

**DOI:** 10.3390/jimaging9100197

**Published:** 2023-09-26

**Authors:** Thittaporn Ganokratanaa, Mahasak Ketcham, Patiyuth Pramkeaw

**Affiliations:** 1 Applied Computer Science Programme, King Mongkut’s University of Technology Thonburi, Bangkok 10140, Thailand; thittaporn.gan@kmutt.ac.th; 2 Department of Information Technology Management, King Mongkut’s University of Technology North Bangkok, Bangkok 10800, Thailand; 3 Media Technology Programme, King Mongkut’s University of Technology Thonburi, Bangkok 10150, Thailand; patiyuth.pra@kmutt.ac.th

**Keywords:** cataract detection, systematic development, LeNet–convolutional neural network

## Abstract

Regular screening and timely treatment play a crucial role in addressing the progression and visual impairment caused by cataracts, the leading cause of blindness in Thailand and many other countries. Despite the potential for prevention and successful treatment, patients often delay seeking medical attention due to the gradual and relatively asymptomatic nature of cataracts. To address this challenge, this research focuses on the identification of cataract abnormalities using image processing techniques and machine learning for preliminary assessment. The LeNet-convolutional neural network (LeNet-CNN) model is employed to train a dataset of digital camera images, and its performance is compared to the support vector machine (SVM) model in categorizing cataract abnormalities. The evaluation demonstrates that the LeNet-CNN model achieves impressive results in the testing phase. It attains an accuracy rate of 96%, exhibiting a sensitivity of 95% for detecting positive cases and a specificity of 96% for accurately identifying negative cases. These outcomes surpass the performance of previous studies in this field. This highlights the accuracy and effectiveness of the proposed approach, particularly the superior performance of LeNet-CNN. By utilizing image processing technology and convolutional neural networks, this research provides an effective tool for initial cataract screening. Patients can independently assess their eye health by capturing self-images, facilitating early intervention and medical consultations. The proposed method holds promise in enhancing the preliminary assessment of cataracts, enabling early detection and timely access to appropriate care.

## 1. Introduction

**Figure 1:**
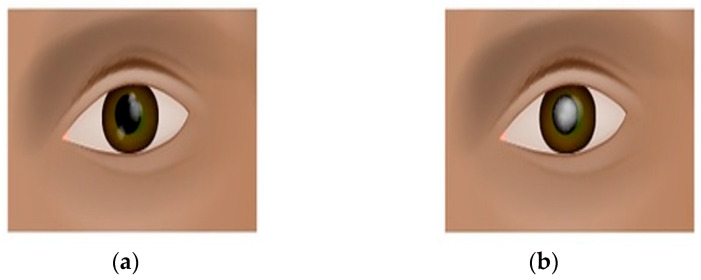
Illustration of eye conditions: (**a**) normal eyes and (**b**) cataracts.

Cataract represents a prevalent ocular disorder characterized by the opacification or clouding of the crystalline lens within the eye. The crystalline lens is an integral component responsible for refracting and focusing incoming light onto the retina, thereby enabling visual perception. Cataracts emerge when this normally transparent and refractive lens undergoes degenerative changes, leading to a loss of transparency and impairment of optical functionality. The etiology of cataracts is multifactorial, encompassing various causative factors. Age-related cataracts, the most common form, develop gradually as a result of intrinsic aging processes, such as oxidative stress and protein denaturation. Additionally, other factors, including genetic predisposition, metabolic disorders (e.g., diabetes mellitus), trauma, long-term exposure to ultraviolet radiation, and certain medications (e.g., corticosteroids) have been implicated in the pathogenesis of cataracts. Clinically, individuals afflicted with cataracts experience a range of visual disturbances that adversely impact their visual acuity and quality of life. The hallmark symptom is a progressive blurring of vision, which gradually worsens over time. As the cataract matures, the affected individuals often report a reduction in visual sharpness and clarity, leading to difficulties in discerning fine details and performing daily activities that require visual precision, such as reading, driving, or recognizing faces. In addition to blurred vision, cataract-related visual impairments encompass alterations in color perception. Colors may appear faded, washed out, or desaturated, hindering the accurate discrimination of hues. Moreover, the presence of cataracts can elicit an increased sensitivity to light, resulting in glare and halos around light sources, especially in low-light conditions or in the presence of bright illumination. Furthermore, individuals suffering from cataracts frequently describe a sensation of visual discomfort, including the perception of darkened or dimmed vision. This reduced contrast sensitivity impairs the ability to distinguish objects against backgrounds of similar tonal value, leading to difficulties in perceiving objects in low-contrast environments. Additionally, cataracts can give rise to visual aberrations, such as the presence of irregularities or lines within the visual field, contributing to visual distortion and further hindrance in accurate visual perception. It is worth noting that cataracts may affect one or both eyes, and the severity and progression of the condition can vary among individuals. Furthermore, the location and extent of lens opacification can vary, ranging from subtle localized changes to widespread clouding of the lens. Given the prevalence of cataracts and their impact on visual function, timely diagnosis and appropriate management are crucial. Cataract surgery, involving the removal of the cloudy lens and its replacement with an intraocular lens, represents the most effective treatment option, offering significant improvements in visual acuity and quality of life for affected individuals. Figure 1 shows the eye conditions of normal eyes and cataracts.

Investigating the unique occurrences of cataracts in the elderly population unveils a deeper understanding of the intricate interplay between aging, ocular health, and visual impairment. This exploration underscores the imperative of a comprehensive and differentiated assessment, enabling a more nuanced approach to diagnosing and treating these specific cataract variations. As we delve into the specific types of cataracts, we encounter nuclear cataracts as the starting point. This variety affects the nucleus, the lens’s central part near the inner corner of the eye. The opacity in this region contributes to cloudiness and a yellow or brown tinge. This distortion not only distorts the passage of light but also hints at the underlying physiological changes occurring within the lens. This type of cataract carries implications beyond mere visual impairment, touching on the complex mechanisms that influence refractive errors and their impact on near and distant vision. It is intriguing how a localized issue can have ramifications that extend beyond the immediate affected area. Transitioning to cortical cataracts, we enter into a realm of lens anatomy that might not be as familiar to the general population. The cortex, a critical component of the lens, undergoes swelling, leading to an opaque appearance. The pattern of clouding, resembling radial grooves, invokes curiosity about the underlying biomechanical processes. It is evident that cataracts are not solely about blurred vision; they offer a glimpse into the intricate molecular changes that unfold within the lens over time. Posterior subcapsular cataracts take us even further, revealing a process occurring around the lens fibers at the back of the lens. The description of “small golden and white grains” opens up a visual representation of the microscopic world within our eyes. This imagery contrasts with our typical perception of the eye as a translucent organ, revealing the complex internal dynamics that contribute to visual acuity and clarity. Understanding these processes adds a layer of complexity to the simple notion of “clouded vision.” What is particularly captivating is the interconnectedness between these cataract variations and other ocular conditions. The link between cataracts and glaucoma, for instance, demonstrates how a single condition can cascade into more severe consequences. The intricate web of cause-and-effect relationships calls for a more holistic approach to diagnosis and treatment, recognizing that eye health is not isolated but part of a broader systemic landscape. By meticulously examining the progression of these cataract variations, we gain insight into the challenges faced by untreated older adults. The cascading effects of abnormalities within the lens are akin to a domino effect, ultimately impacting visual function and overall ocular health. This insight emphasizes the significance of proactive measures, particularly for a demographic that is more susceptible to such disorders. In light of this, the emphasis on early management and treatment becomes not just a medical recommendation but a strategic imperative. It is a call to action to integrate knowledge, awareness, and accessible healthcare to address the specific needs of older adults and mitigate the potential for long-term visual impairment. This in-depth exploration of atypical cataracts and their implications invites us to see beyond the surface of visual impairment and delve into the intricacies of ocular health and aging. It underscores the value of multidisciplinary collaboration and continuous research in enhancing our understanding and management of these complex ocular disorders.

The manifestation of cataract encompasses a perceptible deterioration in visual acuity, characterized by progressive opacification of the eye’s lens. While the initial stages of this ocular condition do not detrimentally impact visual function, its relentless progression eventually impairs visual perception. The incidence of cataracts predominantly afflicts individuals aged 40 years and above [1]. Early identification of cataract pathology holds the potential to avert the exigency for invasive and arduous surgical procedures while concurrently curbing the substantial financial burden. Moreover, timely detection serves as a prophylactic measure against escalating visual debilitation in consonance with the disease’s severity spectrum [2]. The World Health Organization (WHO) [3] reports a staggering global prevalence of approximately 285 million individuals grappling with visual impairment. Among this population, 39 million individuals experience visual constriction, while the remaining fraction endures aberrant visual phenotypes. Notably, cataract pathology assumes responsibility for 33% of visual impairment cases, with an even more daunting statistic of 51% attributing to ocular blindness [4]. In 2020, Flaxman et al. [5] prognosticated the forthcoming figures of individuals afflicted with moderate to severe visual impairment and blindness, approximating 237.1 million and 38.5 million, respectively. Within these prognoses, cataract-induced visual morbidity would impact an estimated 57.1 million individuals (24%) and 13.4 million individuals (35%). The extrapolation warrants the alarming projection that the global population of visually impaired individuals will surpass the 40-million threshold by the year 2025 [6]. Previous research studies have substantiated the minor advancements made in the realm of ocular healthcare and visual impairment management over the past decade. Among the principal etiological factors leading to visual disability, cataract assumes a prominent position [7,8]. The prevailing ocular landscape encompasses three primary categories of cataracts: nuclear cataracts [9], cortical cataracts [10], and posterior subcapsular (PSC) cataracts [11]. These cataract types are commonly attributed to prevalent factors, including advancing age, diabetes mellitus, and tobacco consumption [5]. Early detection of cataracts assumes pivotal significance in mitigating the risk of visual impairment. However, the development of automated cataract detection systems presents formidable challenges, encompassing the intricate interplay of three pivotal aspects: (i) the wavelength regime of cataractous opacities and accompanying color variations, (ii) the morphological dimensions and spatial localization of cataract lesions, and (iii) intrinsic factors, such as the patient’s age, gender, and ocular characteristics. Contemporary investigations have explored an array of imaging modalities as potential candidates for automated cataract detection and classification, including slit-lamp biomicroscopy, retro-illumination imaging, ultrasound imaging, and fundus imaging. In particular, fundus imaging has garnered significant attention in this domain, owing to its facile applicability, even enabling patient self-assessment [12]. In contrast, the utilization of slit-lamp biomicroscopy necessitates the presence of experienced and proficient ophthalmologists, thus impeding its widespread adoption, particularly in developing nations with inadequate healthcare infrastructure [4]. To streamline the intricate landscape of primary cataract screening and facilitate the advent of an automated cataract detection system, a paradigm integrating advanced image processing methodologies is imperative. A multitude of deep learning-based automated cataract detection systems have been documented, predominantly relying on central feature descriptors, such as the Discrete Cosine Transform (DCT) [13], specific local standards, such as local pattern deviation [14], and sophisticated deep convolutional neural networks (CNN) that capitalize on their inherent capacity for profound feature extraction, consequently affording heightened diagnostic accuracy. Nonetheless, despite the extensive proliferation of deep learning-based automated cataract detection systems in the literature, certain inherent limitations persist, encompassing suboptimal detection accuracy, an excessive proliferation of high-dimensional parameters, and computationally intensive operations that engender exorbitant computational costs. Cognizant of the paramount significance and ramifications of timely cataract management, researchers harbor a resolute aspiration to delve into diverse process models that can be harnessed for ocular image processing, thereby empowering patients to conduct preliminary self-assessments of their ocular condition. In this context, researchers have proffered an innovative approach characterized by an image processing workflow tailored for detecting and classifying cataract abnormalities, enabling patients to engage in rudimentary evaluations of their own cataractous manifestations. By enabling preliminary assessments that manifest congruence with visual impairments and somatic distress, this framework imparts patients with the necessary impetus to seek expeditious therapeutic interventions, thereby curbing the trajectory of disease progression and preempting the advent of latent sequelae [4].

The researchers have identified the inherent challenges and profound significance associated with the management of ocular disorders, particularly corneal diseases. Consequently, their primary objective entails a comprehensive investigation of diverse paradigms and methodologies that can be harnessed for the purpose of corneal image analysis. Such endeavors aim to empower patients to engage in preliminary self-assessment of their corneal status, thus facilitating an initial evaluation of their ocular health. To this end, the researchers have meticulously devised a discerning framework, leveraging cutting-edge image processing technologies to discern and classify aberrant corneal conditions. By virtue of this preliminary assessment, complemented by concurrent visual anomalies and corporeal discomfort, affected individuals are prompted to expeditiously seek specialized medical intervention, thereby mitigating the gravity and latent perils that may ensue from deferred therapeutic measures.

The contemporary automated corneal detection systems encompass a tripartite process comprising preprocessing, feature extraction, and classification [15,16,17,18,19,20]. These methodologies can be bifurcated into two cohorts contingent upon the algorithms harnessed for either feature extraction or classification stages: machine learning (ML) and deep learning (DL) paradigms. Recent scholarly investigations have extensively expounded upon these modalities [21,22,23,24]. We aim to succinctly summarize the foremost contributions emanating from both factions.

Machine Learning (ML)-Based Methods: ML-based algorithms have ubiquitously permeated corneal detection systems, serving as the bedrock for extracting pertinent features and effectuating corneal abnormality classification. Prominent studies have harnessed ML techniques, encompassing support vector machines (SVM), random forests (RF), and K-nearest neighbor (KNN) [21]. These endeavors have evinced compelling outcomes in terms of accuracy and efficiency, affording precise identification and taxonomizing of corneal manifestations.

Gao et al. [25] presented a computer-aided corneal detection system designed for large-scale screening and preparatory grading. The system integrates enhanced feature extraction with linear discriminant analysis (LDA) as its primary analytical framework. Experimental evaluations on clinical databases showcase its efficiency, boasting a remarkable accuracy rate of 84.8% in detecting corneal abnormalities.

Yang et al. [26] presented an automated corneal detection methodology utilizing a sequential three-step approach that incorporates the top-bottom hat transformation technique to optimize the contrast between the corneal region and its background. The method considers brightness values and distinctive features during the detection process and employs a backpropagation-based neural network (BBNN) for severity classification, differentiating corneal conditions into mild, moderate, or severe categories. They work on cataracts and highlight a neural network classifier designed for automatic cataract detection in retinal images. This classifier uses enhanced image transformation, feature extraction techniques, and a two-layer BP neural network to grade cataracts by severity. Together, these advancements signify strides in computer-assisted corneal detection and cataract grading, with promising outcomes in diagnostic efficiency and patient care, rooted in techniques such as LDA and BBNN.

Guo et al. [27] introduced a computer-aided corneal categorization system that employs image-based referencing, followed by wavelet-based and morphology-based feature extraction. Subsequently, a multi-level classification algorithm is utilized for corneal detection and grading, yielding correct classification rates (CCRs) of 90.9% and 77.1% for wavelet-based features and 86.1% and 74.0% for morphology-based features, respectively.

Furthermore, Fuadah et al. [28] employed the KNN algorithm for corneal classification and achieved feature fusion by considering image-based characteristics, such as sharpness difference and uniformity. This system was successfully implemented on smartphones, yielding an impressive accuracy rate of 97.5%.

Moreover, a highly competitive corneal detection system was presented by the researchers in [29], where statistical analysis of content-based features was utilized. The system incorporated the Gray-Level Co-occurrence Matrix (GLCM) for content feature extraction. The test dataset was categorized into normal or corneal conditions using KNN, resulting in an average accuracy of 94.5%. It should be noted that manual intervention by users/experts is required during the processing stage, specifically for outlining and segmenting the specific areas of interest. Consequently, the system cannot be considered fully automated. Nevertheless, this system successfully operated on standard eye images captured without employing a slit-lamp or confocal microscopy. These scholarly contributions illustrate significant progress in computer-assisted corneal detection and classification systems. By employing various techniques, such as wavelet transform, image morphology, KNN, and GLCM, these studies have demonstrated promising outcomes in achieving accurate corneal analysis and grading. Consequently, these advancements hold great potential in enhancing diagnostic capabilities and facilitating clinical decision-making processes.

Yang et al. [4] proposed a novel approach for corneal detection and grading using ensemble learning methods. The study involved the extraction of features from distinct feature sets and the construction of dual learning models for each group. By leveraging ensemble methods, such as combining multiple learning models, image categorization was achieved with high accuracy, as evidenced by the correct classification rates (CCRs) of 93.2% for detection and 84.5% for grading.

Similarly, Caixinha et al. [30] developed an automated in vivo system for the detection and categorization of nuclear cataracts. This system incorporated machine learning techniques and ultrasound-based algorithms. By extracting 27 frequency and time-domain features, the researchers explored classification methods, including SVM, Bayes, multilayer perceptron, and random forest, to effectively categorize corneal conditions. Notably, while the ultrasound-based method demonstrated commendable accuracy in corneal screening, it incurred substantial costs and involved a complex image-processing workflow.

Moreover, in [31], a corneal image classification framework utilizing SVM and subsequent grading by an RBF network was presented. This framework achieved a noteworthy specificity rate of 93.33% in assessing the severity of corneal abnormalities. Additionally, Rana and Galib [1] introduced a smartphone application compatible with Android, iOS, and Windows platforms, empowering users to self-diagnose corneal conditions. Through the consideration of texture characteristics and the analysis of relevant data, the application exhibited a commendable accuracy rate of 85%. These investigations underscore the significance of machine learning techniques, ensemble learning, ultrasound-based algorithms, and smartphone applications in the realm of corneal analysis. The integration of these advanced methodologies holds promise for accurate corneal detection, reliable grading, and improved accessibility to corneal diagnostics.

Jagadale et al. [32] proposed the utilization of Hough circle detection transformation as a means to detect the centers and radii of lenses. Subsequently, statistical feature extraction techniques were applied and integrated into an SVM classifier for precise corneal detection, achieving a commendable accuracy rate of 90.25%. Similarly, Sigit et al. [33] presented an innovative approach for corneal detection on Android smartphones. The categorization process was facilitated by a streamlined single perceptron layer methodology, yielding an impressive accuracy of 85%. In a more recent study, the work introduced in [6] introduced a hierarchical feature extraction strategy for corneal grading. The challenge of categorizing corneal severity into four distinct levels was transformed into a more manageable two-level grouping task. This was effectively addressed through the individual training of neural networks, followed by their collective integration. Remarkably, the proposed system achieved detection and grading accuracies of 94.83% and 85.98%, respectively. These research endeavors exemplify the successful application of Hough circle detection, statistical feature extraction techniques, SVM classifiers, perceptron layers, and hierarchical neural networks in the domain of corneal analysis. The adoption of these methodologies has yielded significant advancements in corneal detection, grading, and categorization. Consequently, these findings contribute substantively to the enhancement of corneal diagnostic accuracy and overall efficacy.

Deep Learning (DL)-Based Methods: DL methodologies have attained paramount significance owing to their intrinsic capacity to discern intricate patterns and glean salient representations from intricate datasets. CNNs, an influential subset of DL models, have exhibited extraordinary performance vis-à-vis corneal image analysis [21]. Astute researchers have judiciously employed CNN architectures, such as VGGNet, ResNet, and InceptionNet, to discern and classify corneal aberrations with unprecedented accuracy and resilience. The utilization of deep learning methodologies has demonstrated their efficacy in acquiring essential features and integrating feature learning procedures into the model creation process, thereby mitigating the inherent deficiencies of manual feature engineering and applying them across divergent medical imaging modalities [34,35]. Investigative endeavors by Gao et al. [36] have explored the utilization of deep learning for the categorization of nuclear cataract severity from slit-lamp images. A localized image patch grid was established, whereby image patches were assigned to a CNN to extract regional features. Subsequently, recurrent neural networks (RNNs) were employed to extract increasingly intricate features. The clustering of cataracts was executed utilizing support vector regression (SVR) techniques.

In a similar vein, Zhang et al. [37] proposed a deep convolutional neural network (DCNN) for the detection and grading of cataracts, capitalizing on feature maps derived from hierarchical data fusion structures. This approach proved to be temporally efficient, yielding commendable accuracies in cataract detection and grading, amounting to 93.52% and 86.69%, respectively. These investigations exemplify the successful application of deep learning methodologies in tackling the challenges pertaining to cataract detection and grading. By automating the feature learning process and leveraging advanced neural network architectures, such as CNNs and RNNs, these methodologies offer substantial enhancements in accuracy and efficiency, transcending conventional manual feature engineering techniques. Moreover, the incorporation of SVR and DCNN techniques further elevates the performance and robustness of the models, resulting in promising outcomes in cataract diagnosis and severity assessment. These contributions underscore the potential of deep learning approaches in advancing the realm of medical image analysis and fostering the development of automated systems for ophthalmic healthcare.

Ran et al. [38] developed a novel methodology aimed at accurately grading the severity of cataracts across six distinct levels. The approach employed a synergistic combination of DCNNs and RF to effectively extract and analyze features at various levels from cataract images. Specifically, the methodology consisted of three modules, each contributing to the feature extraction process. The DCNNs were responsible for generating a dataset comprising extracted features, which were subsequently utilized by RF to perform the intricate task of grading cataracts across the complex six-level scale. Notably, the proposed methodology achieved an average accuracy of 90.69%. The multi-level grading system offered by this approach has significant implications for improving the accuracy and precision with which ophthalmology experts comprehend the severity of cataracts in patients. By integrating deep learning techniques with ensemble learning methodologies, Ran et al. [38] have not only achieved high accuracy but have also provided a comprehensive understanding of cataract severity across multiple levels. Such advancements hold considerable potential for enhancing clinical decision-making and ultimately improving the quality of patient care in the field of ophthalmology. In conclusion, the amalgamation of DCNNs and RF in the proposed methodology by Ran et al. [38] represents a notable contribution to the field of cataract severity grading. The rigorous utilization of deep learning and ensemble learning techniques offers a robust framework for effectively analyzing and categorizing cataract images. This research underscores the importance of leveraging advanced computational approaches to address complex medical challenges, thereby paving the way for improved diagnostic accuracy and treatment outcomes.

Pratap and Kokil [39] propose an innovative methodology aimed at facilitating the detection and assessment of cataract severity, ranging from prototype images to advanced stages. Their approach leverages the concept of transfer learning to accomplish automatic cataract classification. The final classification stage is carried out through the utilization of feature extraction techniques in conjunction with SVM. Impressively, the methodology achieves a noteworthy accuracy rate of 92.91% throughout the four-step process. The utilization of transfer learning in this study showcases the potential of leveraging pre-existing knowledge and pre-trained models from prototype images to effectively classify cataracts across varying degrees of severity. By integrating advanced feature extraction methods with SVM-based classification, the proposed approach enables accurate categorization and augments the overall diagnostic capabilities of the system. The reported high accuracy rate exemplifies the efficacy and promise of the proposed methodology in automated cataract classification. Pratap and Kokil’s work significantly contributes to the field of cataract diagnosis and classification by presenting a robust methodology that synergistically combines transfer learning and machine learning techniques. By achieving exceptional accuracy in automated categorization, their approach presents a valuable tool for clinicians and ophthalmologists, empowering them to accurately assess the severity of cataracts. The research demonstrates the potential of leveraging transfer learning to develop automated systems for cataract diagnosis, which ultimately translates into timely and precise treatment decisions for patients. In conclusion, the methodology proposed by Pratap and Kokil [39] underscores the efficacy of transfer learning in automating the classification of cataracts. The amalgamation of sophisticated feature extraction techniques with SVM-based classification underscores the potential of machine learning methodologies in enhancing the diagnostic process for cataract severity assessment. This research opens up new avenues for further advancements in computer-assisted diagnosis and contributes to the collective understanding and management of cataracts within the field.

Jun et al. [40] have presented a pioneering approach in the domain of cataract severity assessment, wherein they introduce a Tournament-based Ranking CNN system. This novel system addresses the critical task of accurately classifying the severity levels of cataracts through the fusion of tournament-based architecture and CNN modeling, thus facilitating robust evaluation of binary labeled cataract data. The Tournament-based Ranking CNN framework leverages the inherent advantages of a tournament structure, enabling comprehensive comparative evaluations of cataract images within the system. By assigning a ranking to each image based on its severity level, the framework establishes a reliable hierarchy that captures the varying degrees of cataract severity. Concurrently, the CNN model integrated within the framework plays a pivotal role in extracting salient features from the cataract images, contributing to precise classification outcomes. The adoption of a binary labeled dataset in this study empowers the system to discriminate between normal and cataract-affected images with remarkable efficacy. The incorporation of the Tournament-based Ranking CNN methodology marks a significant milestone in the field of cataract severity assessment, revolutionizing the traditional approaches prevalent in clinical settings. The system’s ability to rank cataract images based on their severity not only offers quantitative metrics but also augments the decision-making process for clinicians, facilitating tailored treatment strategies. Furthermore, the integration of CNN-based feature extraction mechanisms enhances the system’s discriminative capabilities by capturing high-level characteristics indicative of cataract severity. The research conducted by Jun et al. establishes a notable contribution to the expanding body of knowledge in the field of cataract diagnosis and severity assessment. The Tournament-based Ranking CNN system presents a robust and efficient methodology for objectively evaluating cataract severity levels. The utilization of binary labeled data, coupled with the incorporation of CNN-based feature extraction, accentuates the system’s precision, thereby advancing the accuracy and reliability of cataract severity classification. To summarize, Jun et al. [40] introduce an innovative system in their research, encompassing the Tournament-based Ranking CNN framework for cataract severity classification. This cutting-edge methodology amalgamates tournament-based evaluations and CNN modeling, enabling accurate ranking and classification of cataract images according to their severity levels. The findings demonstrate promising outcomes, promising to automate and streamline cataract severity assessment, empowering clinicians with vital information for informed decision-making and optimal patient care.

Hossain et al. [41] have made notable contributions to the field of cataract detection by proposing a sophisticated system that leverages DCNNs and Residual Networks (ResNets). Their work focuses on achieving high accuracy in categorizing cataract severity levels. In a similar vein, Zhang et al. [42] have presented a cataract detection method that employs an ensemble approach, incorporating ultrasound images to enhance accuracy, which surpasses alternative deep learning methodologies with a remarkable 97.5% precision. The proposed system comprises three key components: an object detection network, multiple categorization networks, and a model fusion module. Performance evaluation of the system demonstrates satisfactory outcomes, particularly in scenarios with limited training data. However, it is important to acknowledge a primary limitation of this methodology, namely its reliance on image clarity as a reference for assessing cataract severity, as this criterion may be influenced by other ocular conditions such as corneal opacities and diabetic retinopathy. Consequently, the method may not be adept at distinguishing between different types of ocular diseases. The research conducted by Hossain et al. [41] and Zhang et al. [42] represents a significant advancement in cataract detection and severity assessment. The utilization of DCNNs, ResNets, and the ensemble methodology underscores their effectiveness in accurately categorizing cataracts. These findings hold great promise for enhancing the diagnostic process and assisting healthcare professionals in making informed decisions regarding patient care. Nonetheless, future investigations should address the challenges associated with classifying various ocular conditions and explore novel techniques to further enhance the overall performance and reliability of the system. In summary, the research conducted by Hossain et al. [41] and Zhang et al. [42] constitutes a notable contribution to the field of cataract detection. Their utilization of advanced deep learning architectures, such as DCNNs, ResNets, and the ensemble methodology, demonstrates the potential to achieve accurate categorization of cataract severity. However, it is essential to acknowledge the limitations imposed by the reliance on image clarity as a criterion for severity assessment, as this may hinder the differentiation of different ocular diseases. Further research efforts in this domain have the potential to refine cataract diagnosis and significantly impact patient outcomes.

Khan et al. [43] conducted a comprehensive investigation into the measurement of cataract severity from panoramic dental images. Their study employed the VGG-19 model with transfer learning techniques on the latest publicly available dataset from the KAGGLE platform [44]. Remarkably, their approach achieved a near-equivalent accuracy rate of 97.47%, establishing a new benchmark in cataract detection. In a related work, Pratap and Kokil [45] published a novel method for aiding cataract diagnosis in environments plagued by disruptive noise. By leveraging a dedicated CNN trained on both internal and external datasets, they demonstrated the resilience of their approach even under adverse conditions. This pioneering research represents a pioneering exploration into the robustness of cataract detection systems, particularly in the presence of acoustic interferences. The contributions made by Khan et al. [43] and Pratap and Kokil [45] constitute notable advancements in the field of cataract detection. The utilization of the VGG-19 model and dedicated CNN architectures in these studies attests to their efficacy in achieving highly accurate cataract diagnosis. Moreover, the investigation of system resilience to environmental noise holds substantial potential for enhancing the reliability and practicality of cataract detection methodologies. Further validation and refinement of these approaches within real-world clinical settings will yield valuable insights into their integration into the existing healthcare framework. In summary, the research conducted by Khan et al. [43] and Pratap and Kokil [45] significantly contributes to the domain of cataract detection. Their utilization of advanced models, such as VGG-19, along with tailored CNN architectures, highlights the potential for achieving exceptional accuracy in cataract diagnosis. Furthermore, the exploration of system robustness in the presence of environmental noise demonstrates a commendable effort to address practical challenges faced in clinical settings. Ongoing research endeavors in this area are expected to further enhance cataract detection methodologies, ultimately impacting clinical decision-making processes in a meaningful manner.

Observations reveal a conspicuous disparity [46,47,48] between the abundance of research endeavors following conventional machine learning paradigms and the paucity of investigations reporting on cataract detection and severity classification through deep learning methodologies. Consequently, a multitude of obstacles persist, necessitating diligent attention. Paramount among these challenges is the imperative to heighten model precision whilst concurrently mitigating complexity by curtailing the number of parameters during the training phase, the stratum count, the depth, the runtime, and the overall dimensions of the model. To augment the accuracy of deep learning models for cataract detection and severity classification, the employment of sophisticated regularization techniques emerges as a promising avenue. By incorporating regularization mechanisms such as dropout, L1 or L2 regularization, or batch normalization, models can effectively assuage overfitting and bolster generalization prowess. Moreover, the exploration of advanced optimization algorithms, including Adam, RMSprop, or learning rate scheduling, holds the potential to fortify model convergence and overall performance. Additionally, harnessing cutting-edge architectures like ResNet, DenseNet, or Inception offers prospects for refining feature representation and hierarchical learning, facilitating the discernment of nuanced patterns and intricate relationships embedded within cataract images. Fine-tuning pre-trained models on voluminous and heterogeneous datasets can further surmount limitations attributable to inadequate training samples, thus culminating in elevated performance and heightened generalizability [49,50,51]. Furthermore, grappling with the exigencies associated with interpretability and explainability in deep learning models tailored to cataract detection assumes paramount significance. The development of techniques enabling visualization and interpretation of learned features and decision-making processes in these models engenders profound insights into their inferential outputs, fostering trust and facilitating their adoption within clinical milieus. To recapitulate, despite the extensive examination of traditional machine learning approaches in cataract detection, an array of challenges intrinsic to deep learning methodologies remains extant. Enhanced model accuracy while minimizing complexity, the adept utilization of advanced regularization and optimization techniques, the exploration of avant-garde architectures, and the resolution of interpretability quandaries all constitute pivotal domains warranting sustained scrutiny [52,53,54,55]. By surmounting these impediments, the field can ascend towards the realm of more precise, efficient, and explicable deep learning models for cataract detection and severity classification.

Santra et al. [56] presented a novel research endeavor focused on the development of an automatic classification system for optic nerve diseases. Employing sophisticated computer vision algorithms, the study explored two distinctive approaches to tackle this problem. The first approach encompassed a traditional computer vision methodology, which was subsequently complemented by the integration of a machine learning (ML) model. Various ML algorithms, namely multinomial logistic regression, random forest, gradient boosting classifier, and support vector machine, were employed to train the data and extract valuable insights from the optic nerve images. The second approach, characterized by automatic feature engineering and classification, leveraged CNN. This technique facilitated the creation of multiple class-specific models, enabling the accurate identification of specific diseases as well as binary classification to determine disease presence or absence. To evaluate the performance of the developed models, a carefully labeled dataset comprising 600 retinal images was utilized for training purposes. Results indicated that the ML models exhibited remarkable accuracy, surpassing 90% in correctly identifying various optic nerve diseases. Conversely, the CNN models yielded less satisfactory outcomes during the testing phase, with an accuracy below 60%. Accuracy was calculated as the ratio of correctly classified images to the total number of images within the dataset. These findings underscore the efficacy of the ML approach in effectively discerning optic nerve diseases, thus highlighting its potential for clinical application. However, further refinement and optimization of the CNN models are warranted to enhance their accuracy and reliability. The work conducted by Santra et al. represents a significant contribution to the field of automatic classification of optic nerve diseases, opening avenues for future research and innovation in this domain. This study [57] addresses challenging hemorrhage segmentation in retinal images, particularly on mobile phones, with poor lighting. A novel KMMRC-INRG method enhances segmentation by addressing uneven illumination using KMMRC and improving boundary segmentation through INRG. The approach achieves high performance, with recall, precision, F1 measure, and IoU scores of 80.18%, 91.26%, 85.36%, and 80.08%, respectively. Notably, the F1 measure improves by 19.02% compared to DT-HSVE on the same dataset and up to 58.88% for images with large hemorrhages.

## 2. Materials and Methods

**Figure 2:**
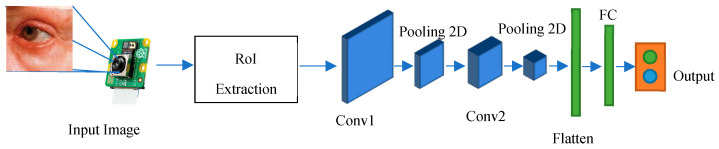
Overview framework of the proposed method.

The current research endeavors reflect a considerable body of work focusing on the adoption of CNNs as a deep learning framework for the detection and severity assessment of glass fractures. CNNs are characterized by their utilization of convolutional and pooling operations across different layers, accompanied by non-linear activation functions, to establish a hierarchical feature representation. Notably, the integration of feature extraction and classification processes within the deep learning paradigm distinguishes this approach from conventional handcrafted feature extraction methods, which often treat these steps as distinct entities. Nevertheless, the investigation of deep learning-based approaches for glass fracture detection and severity assessment remains relatively limited compared to the extensive exploration of traditional machine learning techniques. To address this gap, the current study introduces a novel methodology that leverages convolutional neural networks, specifically a variant known as LeNet, to overcome the constraints associated with handcrafted feature extraction and computational complexity. LeNet, originally popularized as Lenet-5, is a specific instantiation of a feed-forward neural network architecture that excels in processing large-scale images, wherein individual neurons exhibit receptive fields and demonstrate remarkable performance within their local contexts. In the context of the present research, the LeNet-5 variant has been specifically tailored to accommodate the demands of glass fracture detection. Consequently, it encompasses a convolutional neural network comprising seven distinct layers, each contributing to the processing pipeline, and effectively operates on digital images with dimensions of 32 × 32 pixels. The adoption of LeNet within the research framework signifies its efficacy in overcoming the limitations associated with traditional handcrafted feature extraction techniques while simultaneously mitigating the computational complexity inherent in processing high-resolution images. By embracing the convolutional neural network paradigm, the study capitalizes on the innate strengths of deep learning models, ultimately facilitating enhanced performance and the generation of efficient feature representations. Methodologically, the research endeavor encompasses a systematic workflow with various stages. These stages involve comprehensive data preprocessing, the thoughtful design of network architecture, meticulous model training utilizing backpropagation, and rigorous model evaluation employing appropriate performance metrics. Importantly, the iterative nature of deep learning methodologies enables the refinement of models across multiple training epochs, allowing for the optimization of network weights and biases and the subsequent enhancement of predictive capabilities. To summarize, the present research contributes to the existing body of knowledge by integrating convolutional neural networks, specifically the LeNet architecture, within the deep learning paradigm to tackle the challenges associated with handcrafted feature extraction and computational complexity. Through the proposed methodology, the study highlights the potential of deep learning approaches in effectively processing high-resolution images and provides insights into the sequential stages involved in implementing the research framework as shown in Figure 2.

The architecture of the implemented system involves the use of a CNN for classifying eye images into two categories: those with cataracts and those without. The architecture consists of several essential layers. Convolutional Layers: These layers are responsible for learning important features from the input images through convolution operations, which help in identifying patterns like edges, corners, and shapes. Activation Functions (ReLU): Rectified Linear Activation functions introduce non-linearity to the model, enhancing its capability to capture complex relationships in data. Max-pooling Layers: These layers reduce the dimensions of the feature maps, thus reducing computational load and focusing on the most relevant information. Flatten Layer: This layer converts the matrix-like feature maps into a single vector, preparing the data for fully connected layers. Fully Connected Layers: These layers process the extracted features and make class predictions based on the learned representations.

The training process involves several steps: (i) Data Loading: The dataset is loaded, resized, and preprocessed to be compatible with the CNN model. (ii) Data Splitting: The dataset is divided into training and testing subsets for training and evaluation purposes. (iii) Data Encoding: Labels are encoded into numerical format using techniques like LabelEncoder and OneHotEncoder. (iv) Model Compilation: The CNN model is built using Keras Sequential API, incorporating convolutional, activation, max pooling, and fully connected layers. (v) Optimizer and Loss Function: Stochastic Gradient Descent (SGD) is selected as the optimizer, and binary cross-entropy is chosen as the loss function due to the binary classification nature of the problem. (vi) Model Training: The model is trained on the training data using the defined optimizer and loss function, with a specified number of epochs and batch size. (vii) Evaluation: The trained model is evaluated on the testing data to assess its performance using metrics such as accuracy and classification reports.

Hyperparameter settings include the learning rate (lr), which is set to 0.1 in the SGD optimizer, controlling the step size during weight updates. We set momentum to 0.0, affecting the weights’ update direction based on past updates. Decay is set to 0.0, controlling the learning rate decay during training. Batch size is set to 150, determining the number of samples processed before updating the model. Number of epochs is set to 50, representing the number of times the entire training dataset is passed through the model during training.

### 2.1. Preprocessing

**Figure 3:**
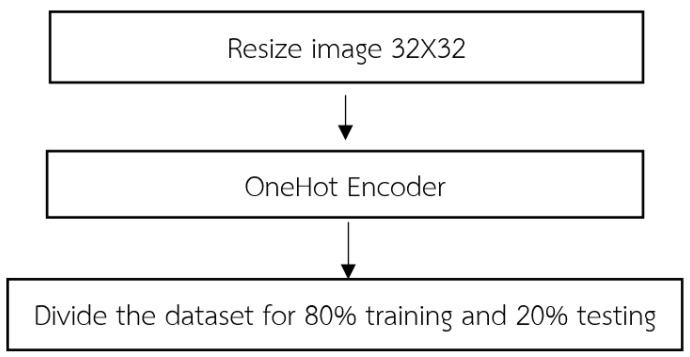
Preprocessing procedure of LeNet-CNN.

Upon the initiation of data importation, the acquired dataset undergoes a fundamental stage known as data preprocessing. This pivotal process encompasses a series of methodical steps aimed at refining and organizing the dataset to ensure its suitability for subsequent analyses as shown in Figure 3. The following steps typically constitute.

(1) In order to achieve uniformity and compatibility across the dataset, a crucial preprocessing step involves resizing the input images to a consistent dimension of 32 × 32. This resizing process is integral to the LeNet-CNN architecture and involves two primary steps: the convolutional layer and the pooling layer. For the convolutional layer, this layer employs convolutional filters to perform localized receptive field operations on the input images. By convolving the filters across the image, relevant features are extracted while simultaneously reducing the spatial dimensions of the image. This process plays a pivotal role in adjusting the image size to conform to the desired dimensions. Following the convolutional layer, the pooling layer further contributes to the resizing process. Utilizing techniques such as max pooling or average pooling, this layer reduces the dimensionality of the convolved feature maps. By aggregating information from neighboring regions, the pooling layer effectively downsamples the feature maps, resulting in images of the desired size. By sequentially applying these two fundamental steps within the LeNet-CNN architecture, the imported dataset undergoes a comprehensive resizing procedure, yielding images with a standardized dimension of 32 × 32. This standardized image size ensures consistency and facilitates subsequent stages of analysis, feature extraction, and classification.

The purpose of encoding 32 × 32 images is primarily to maintain uniformity and compatibility throughout a dataset, especially when using architectures such as the LeNet-CNN. This consistency ensures smooth data flow, optimal feature extraction, and efficient model training and performance. The LeNet-CNN architecture is designed to work optimally with 32 × 32 images, considered to be standard dimension for the model to perform efficiently. Specifically, the convolutional layer, the first step in adjusting the image size, uses filters to scan the image and capture localized features. As these filters move across the image, they reduce its spatial dimensions while preserving and emphasizing relevant features, bringing it closer to the desired 32 × 32 size. Following the convolutional process, the pooling layer further aids in achieving the desired image size. It simplifies the feature maps by condensing information. The goal is to obtain an abstracted yet informative representation of the original image. Once images are resized to a consistent 32 × 32 dimension, it becomes easier to apply further analysis, feature extraction, and classification operations. This standardization ensures that the entire dataset can be processed uniformly, reducing errors and complications that may arise from inconsistent image dimensions. In essence, encoding 32 × 32 images provides a consistent foundation for data processing, especially when using architectures like LeNet-CNN.

**Figure 4:**
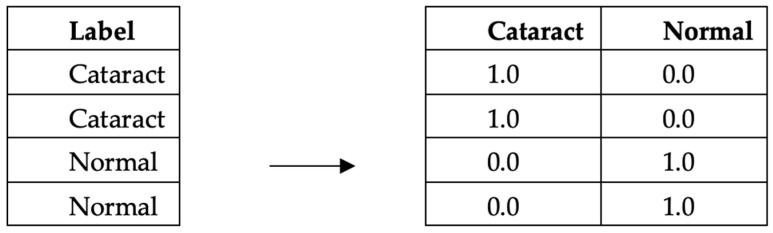
Example of encoding data type OneHotEncoder.

- The convolutional layer within the LeNet-CNN architecture utilizes small-sized convolutional filters to extract relevant features from input images. During the convolutional operation, the image undergoes pixel-wise multiplication with the weight values present in the convolutional filters. This process yields a new feature map that represents the extracted features. The filters are slid across the image, applying the convolution operation by using a predefined stride value. By convolving the filters, the convolutional layer effectively extracts important visual patterns and characteristics of the image, which are subsequently utilized in subsequent stages of the network. This process of convolution plays a vital role in feature extraction and enables the network to capture spatial dependencies and patterns within the image.
- The pooling layer is employed in the LeNet-CNN architecture to reduce the dimensionality of the feature maps obtained from the convolutional layer. In LeNet-CNN, the max-pooling technique is utilized, which selects the maximum value from a specified group of pixels within the feature map. The kernel size is defined to determine the size of the pixel group, and the stride value is employed to specify the displacement when moving to the next set of pixels. The pooling operation helps decrease the size of the feature maps while preserving important features. By reducing the dimensionality, the pooling layer aids in controlling the computational complexity of the network and achieving translation invariance, allowing the network to focus on the most salient features and discard redundant information.

(2) OneHot encoding is a data processing technique employed in image data to transform categorical labels into numerical representations for computational purposes. This method encodes each label as an independent binary vector, enabling the creation of machine learning models that exhibit enhanced accuracy and performance. OneHot encoding is used to convert categorical variables into a numerical format suitable for consumption by machine learning models. The underlying principle of OneHot encoding involves transforming each category value into a vector of equal length, representing all the categories as shown in Figure 4. Each position in the vector corresponds to a specific category, with a value of 1 indicating the presence of the variable in that category and a value of 0 indicating its absence. By representing categorical variables as binary vectors, OneHot encoding facilitates the utilization of these variables in machine learning algorithms, enabling effective model training and inference. We apply this encoding to the labels in both the training and the test datasets. This ensures that the model’s output can be correctly matched and evaluated against the true labels.

(3) The dataset is divided into training and testing sets, following the conventional practice of an 80–20% split. This partitioning scheme ensures that 80% of the data are allocated for model training while the remaining 20% is reserved for evaluation purposes. By separating the dataset into these distinct subsets, we can effectively assess the performance and generalization capabilities of the trained model on unseen data. The training set serves as the foundation for model learning, enabling the optimization of model parameters through iterative processes such as gradient descent. On the other hand, the testing set serves as an unbiased evaluation metric, allowing us to gauge the model’s performance on unseen data and estimate its ability to generalize to new instances. This division strategy helps mitigate issues related to overfitting, as the model is assessed on independent data that it has not been exposed to during the training phase. Consequently, the 80–20% split facilitates robust model development and evaluation in a rigorous and statistically sound manner.

### 2.2. Define the Layer of the CNN

**Figure 5:**
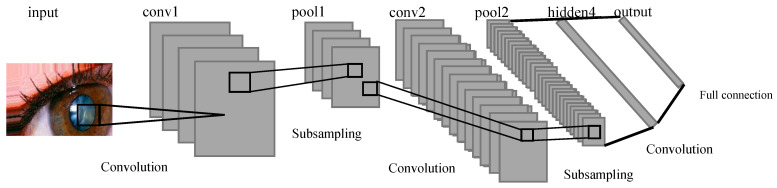
Illustration of the layers within LeNet-CNN.

In the CNN architecture, the composition of layers can be defined to incorporate hidden layers and the classification layer as shown in Figure 5. In the initial layer, a convolutional layer is created with a filter/kernel size of 3 × 3. The 3 × 3 filter/kernel is applied elementwise to the first 3 × 3 patch of the image, and the resulting values are summed. The sum is then placed in the first row, first column of the output matrix. Next, the 3 × 3 filter/kernel is shifted one position to the right in the first matrix, and the process is repeated. The resulting values are placed in the next column of the output matrix. This process continues until the entire output matrix is filled. The number of filters output is set to 32. Padding is applied to ensure that the size of the output matrix matches the size of the original image. The activation function used is ReLU, which helps in faster training of the model. This configuration of layers enables the CNN to perform feature extraction on the input data and learn meaningful representations that can be used for classification tasks. The convolutional layer with 3 × 3 filters performs local feature detection, while the ReLU activation function introduces non-linearity to enhance the model’s expressive power. The padding ensures that the spatial information is preserved during the convolutional operation. The resulting feature maps from this layer can be further processed by subsequent layers, such as pooling layers and fully connected layers, to extract higher-level features and make predictions for classification.

(1) $$f\left(x\right)=\max\left(0,x\right)=\left\{\begin{array}{c} 0\;\mathrm{for}x\leq 0\\ x\;\mathrm{for}x>0 \end{array}\right.$$

Furthermore, a max-pooling layer with a size of 2 × 2 is incorporated into the CNN architecture. Max pooling aims to extract the most important parts of the data while increasing the efficiency of processing. It achieves this by selecting only the maximum values within a grid and storing them in the second layer. Similar to the previous layer, a 3 × 3 convolutional layer is defined with padding and ReLU activation function as shown in Equation (1). Additionally, a 2 × 2 max pooling is applied. The number of filters output remains the same at 64.

Moving on to the third layer, it consists of a fully connected layer, which is connected to every node in the previous layer. However, since the data are now in a 1-dimensional form, a process called flattening is performed to convert the data from its original 3-dimensional shape to a 1-dimensional form. The flattened data are then connected to a dense layer with a size of 512, followed by the ReLU activation function.

In the fourth layer, the results obtained from the previous layers are fed into the SoftMax classifier. This final layer enables the model to perform the subsequent classification task.

In summary, the CNN architecture involves the following steps: convolution layer (3 × 3) with padding and ReLU activation, max-pooling layer (2 × 2), another convolution layer (3 × 3) with padding and ReLU activation, max-pooling layer (2 × 2), and a fully connected layer with flattening, dense layer (512) with ReLU activation. The output of these layers is then passed through the SoftMax classifier for classification purposes.

1. Input Layer: The first step in the LeNet-CNN architecture is the input layer, which receives small-sized images with dimensions of 32 × 32. These images serve as the input data for the network;
2. Convolutional Layer: The second step involves using filters or kernels to perform convolutions on the input images. This process generates feature maps that capture spatial features of the images, such as detecting edges or important patterns. In LeNet-CNN, there are typically multiple convolutional layers that extract specific image features;
3. Pooling Layer: The third step employs pooling techniques to reduce the size of the feature maps and reduce the complexity of the data before passing it to the next layer. LeNet-CNN utilizes max pooling, which selects the maximum value within a small, specified region of the feature maps;
4. Fully Connected Layer: This layer is responsible for connecting to the feature maps that have undergone convolution and pooling. It consists of fully efficient cells capable of learning patterns and relationships within the data. It resembles the dense layer of an artificial neural network but with spatial limitations;
5. Output Layer: The final step is the output layer, which has the same number of cells as the desired classification classes. Each cell provides OneHot encoded output, indicating the probability of the image belonging to each class.

In summary, the LeNet-CNN architecture involves an input layer, convolutional layers for feature extraction, pooling layers for downsampling, fully connected layers for pattern learning, and an output layer for classification.

### 2.3. Model Training

After defining the layers of the CNN, the model can be trained by setting the following parameters:

1. Parameter setting

**Figure 6:**
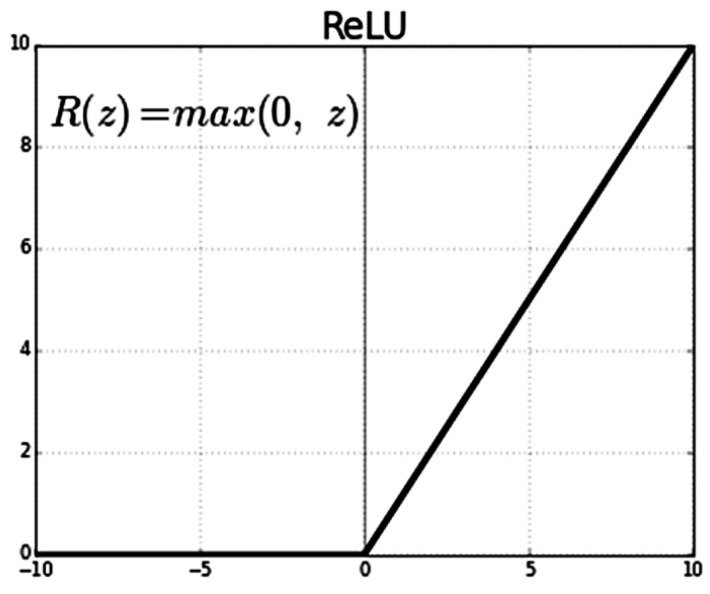
ReLU activation function.

**Figure 7:**
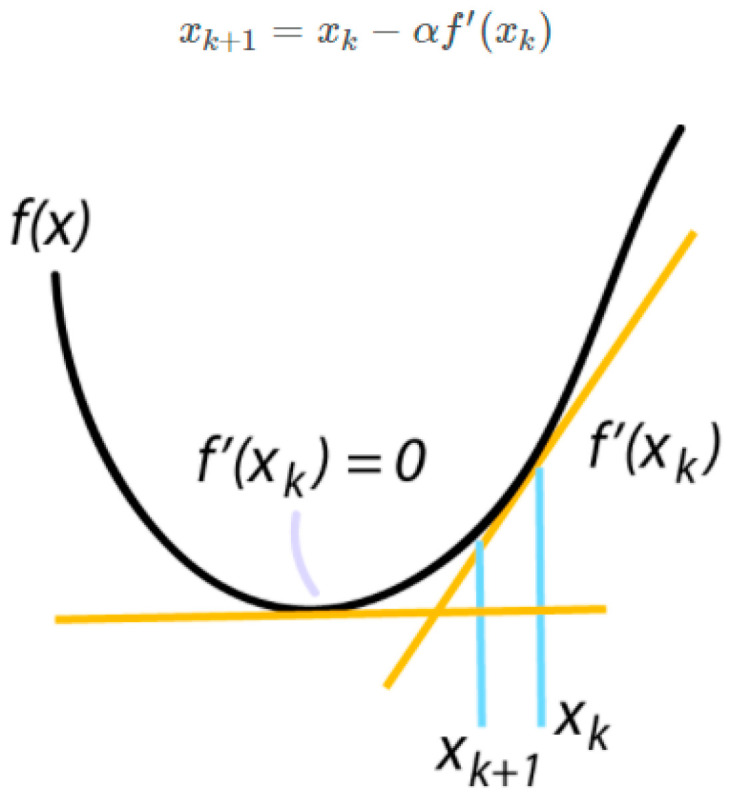
Equation for finding the lowest point of the area of SGD.

We set the parameters of the model as follows:

- Batch Size: 16. It represents the number of data samples that will be propagated through the network in each training iteration;
- Epochs: 25. It indicates the number of complete passes the model will make through the training dataset during training;
- Learning Rate: 0.1. It determines the step size at which the model’s weights are updated during training;
- Loss Function: binary cross-entropy. It is the average value of the cross-entropy between the actual and predicted probability distributions of two classes (Class 0 and Class 1). We also use Relu as the activation function as shown in Figure 6. A lower average value is preferable, and the binary cross-entropy loss is defined as follows:

In this work, we use binary cross-entropy loss function, which is often referred to as log loss or logistic loss. It is a commonly used loss function in binary classification tasks, where the goal is to predict one of two possible outcomes (usually 0 or 1). It measures the difference between predicted probabilities and actual binary labels. The formula for binary cross-entropy loss is shown in Equation (2),

(2) $$Loss=-\frac{1}{output\;size}\sum_{i=1}^{output\;size}y_{i}\cdot \log{\hat{y}}_{i}+(1-y_{i})\cdot \log\left(1-{\hat{y}}_{i}\right)$$

where:

- *Loss* is the binary cross-entropy loss for a single data point;
- *y* is the actual binary label (either 0 or 1);
- *ŷ* is the predicted probability of the positive class (class 1) by the model.

2. Training the model:

During training process, we first create training and testing datasets. Then, we iterate through the specified number of training epochs. The training dataset is divided into batches according to the specified batch size. We then feed each batch of data through the CNN model and calculate the loss value using the binary cross-entropy loss function. We use the gradient descent algorithm optimizer, Stochastic Gradient Descent (SGD), to adjust the parameter values in the model, improving the model efficiency. The gradient provides information about the size and direction of parameter updates, guiding the loss value towards the minimum point on the surface. Then, we iteratively adjust the parameter values in each training iteration until the specified number of training epochs is reached. The equation for finding the lowest point of the area of SGD is shown in Figure 7.

For the model evaluation, we utilize the test dataset to evaluate the trained model by calculating performance metrics such as accuracy, precision, recall, F1 score, or error rate. After training the model, it is important to assess its performance using the test dataset. The test dataset contains data that the model has not seen during training and serves as an unbiased evaluation of its generalization capability. Performance metrics such as accuracy, precision, recall, F1 score, or error rate are computed to measure how well the model predicts the correct class labels.

For iterative training and evaluation, we repeat the training and evaluation steps for the specified number of training iterations. In order to improve the model’s performance, the training and evaluation steps are iteratively repeated for the designated number of training iterations. This process allows the model to learn from the data, update its parameters, and improve its predictions over time. By iteratively training and evaluating the model, it has the opportunity to learn and adapt to the patterns and complexities present in the dataset. After completing the training and evaluation iterations, the results are analyzed and summarized.

3. Evaluation metrics

The evaluation metrics and statistics utilized are as follows:

- True positive (TP) signifies the number of instances that are accurately predicted as positive with respect to the positive class;
- False positive (FP) indicates the number of instances that are erroneously predicted as positive when compared to the positive class;
- True negative (TN) denotes the number of instances that are correctly predicted as negative with respect to the negative class.
- False negative (FN) represents the number of instances that are incorrectly predicted as negative when compared to the negative class;
- N represents the total number of data points within the dataset.

These metrics serve as crucial measures for evaluating the model’s performance and its ability to accurately classify instances into positive and negative classes. By analyzing these metrics, one can assess the model’s accuracy, precision, recall, and F1 score, all of which are fundamental indicators of its efficacy in addressing the given task. The accuracy metric provides information about the percentage of instances that the model correctly predicts. It is calculated as the ratio of the sum of true positive (TP) and true negative (TN) to the total number of data points (N). The accuracy metric ranges from 0 to 1, where a value closer to 1 indicates that the model’s predictions are highly accurate. The accuracy can be computed as shown in Equation (3).

Accuracy = (TP + TN)/N(3)

Precision is a metric that measures the accuracy of a model in predicting the relevant instances in the positive class. It is calculated by dividing the number of true positive predictions (TP) by the sum of true positive predictions and false positive predictions (TP + FP). The value of precision ranges from 0 to 1, where a higher precision value indicates a higher level of accuracy in predicting the positive class. The precision can be computed as shown in Equation (4).

Precision = TP/(TP + FP)(4)

Recall, also known as sensitivity or true positive rate, is a metric that measures the accuracy of a model in predicting the occurrences of a specific event in the positive class. It is calculated by dividing the number of true positive predictions (TP) by the sum of true positive predictions and false negative predictions. The recall can be computed as Equation (5):

Recall = TP/(TP + FN)(5)

F1 score is the harmonic mean of precision and recall. It is a metric that provides a balanced measure of a model’s performance by taking into account both precision and recall simultaneously. The F1 score is calculated by taking the reciprocal of the arithmetic mean of the reciprocals of precision and recall. F1 score combines the precision and recall values into a single metric, allowing us to evaluate the overall effectiveness of a model in terms of both correctly predicting positive instances (precision) and capturing all actual positive instances (recall). It provides a consolidated measure that balances the trade-off between precision and recall. The F1 score ranges from 0 to 1, where a value closer to 1 indicates a better balance between precision and recall and reflects a more accurate and reliable model performance. It is particularly useful when the data are imbalanced or when both precision and recall are equally important in the evaluation of the model’s effectiveness. The F1 score can be computed as shown in Equation (6).

F1 = 2TP/(2TP + FP + FN)(6)

True negative rate, also known as specificity, is a metric that measures the model’s ability to correctly predict negative instances or non-disease conditions. Specifically, it represents the proportion of actual negative instances that are correctly identified as negative by the model.

Specificity is an important performance measure in binary classification problems, particularly when the negative class or non-disease condition is of interest. It quantifies the model’s ability to discriminate and classify negative instances accurately, minimizing the occurrence of false positives. A higher specificity value indicates that the model is proficient in identifying true negative instances and has a lower tendency to misclassify non-disease conditions as positive. It signifies the model’s capability to minimize the occurrence of Type I errors, which are false positive predictions. Specificity is calculated as the ratio of true negatives (TN) to the sum of true negatives and false positives (TN + FP). It ranges from 0 to 1, where a value closer to 1 signifies a higher level of accuracy in predicting negative instances and a better ability to distinguish non-disease conditions correctly. The calculation of specificity is shown in Equation (7).

TNR = TN/(TN + FP)(7)

## 3. Results

### 3.1. Datasets

**Figure 8:**
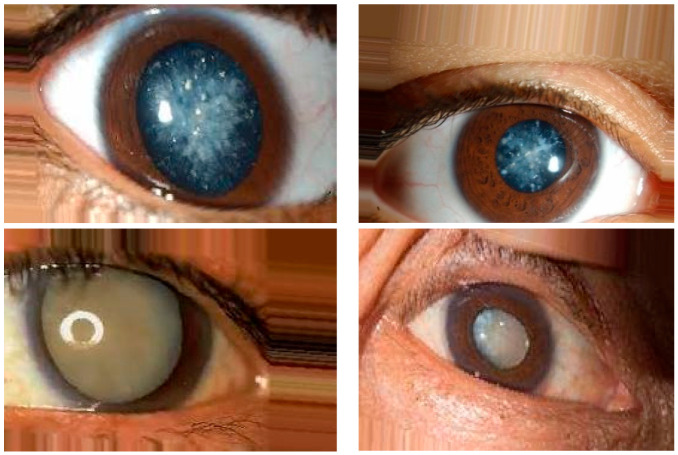
Image examples of eyes with cataract symptoms.

**Figure 9:**
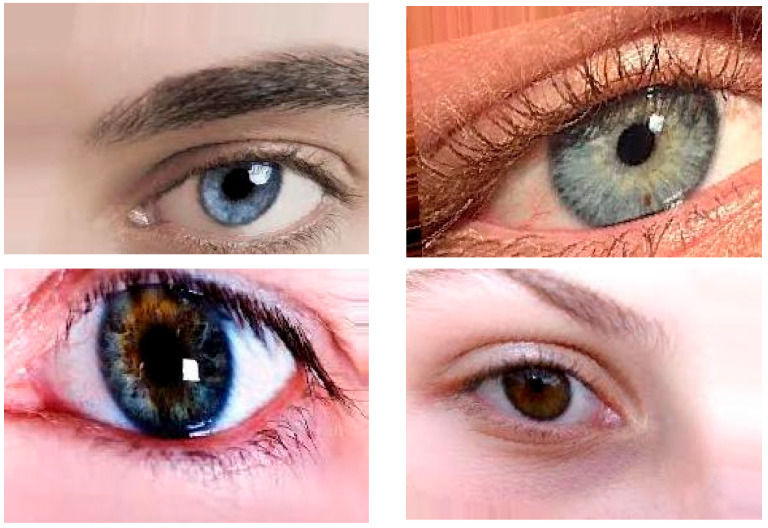
Image examples of normal eye conditions.

In this research, publicly available datasets [58,59] were utilized. The datasets consisted of 3500 images of eyes displaying symptoms of glaucoma and 3500 images of normal eyes. The datasets were divided into a training set, comprising 80% of the data, and a testing set, comprising the remaining 20%. The dataset used in this study was obtained from public websites [58,59]. It consisted of a collection of 3500 images showing symptoms of glaucoma and an equal number of images of normal eyes. The dataset was split into an 80% training set and a 20% testing set for the purpose of model training and evaluation. The dataset employed in this investigation was sourced from publicly accessible websites [58,59]. It encompassed a total of 3500 images portraying the manifestation of glaucoma symptoms and an equivalent number of images depicting normal eyes. To facilitate the training and evaluation process, the dataset was partitioned into an 80% training subset and a 20% testing subset. For this research, a publicly available dataset [58,59] was utilized. The dataset comprised 3500 images displaying symptoms of glaucoma and an equal number of images depicting normal eyes. To train the model, 80% of the data were used as the training set, while the remaining 20% served as the testing set for evaluation purposes. The image examples of eyes with cataract symptoms and normal eye conditions are shown in Figure 8 and Figure 9, respectively.

### 3.2. Convolutional Neural Network Testing Results

Initially, CNN was trained with parameter values set as follows: batch size of 128, 25 epochs, learning rate of 0.1, binary cross-entropy loss function, and Stochastic Gradient Descent (SGD) optimizer. The testing results showed an accuracy value of 0.94. The initial testing of the CNN was conducted with specific parameter values. The batch size was set to 128, the number of epochs was 25, the learning rate was 0.1, the loss function used was binary cross-entropy, and the optimizer employed was Stochastic Gradient Descent (SGD). The obtained result yielded an accuracy value of 0.94. The CNN was initially trained and tested with a specific set of parameters. The batch size was set to 128, the number of epochs was 25, the learning rate was 0.1, the loss function used was binary cross-entropy, and the optimizer employed was SGD. The testing results showed an accuracy of 0.94. During the initial testing phase, the CNN was trained using specific parameter configurations. The batch size was set to 128, the number of epochs was 25, the learning rate was 0.1, the loss function was binary cross-entropy, and the optimizer used was SGD. The achieved accuracy for the testing phase was 0.94 as shown in Table 1.

The CNN achieved a high level of performance in classifying eye images with cataract symptoms, correctly identifying 670 images. Moreover, it demonstrated proficiency in distinguishing normal eye images, accurately classifying 641 samples. However, the model exhibited some misclassifications, as it erroneously labeled 31 images with cataract symptoms as normal and 58 normal eye images as showing cataract symptoms.

**Figure 10:**
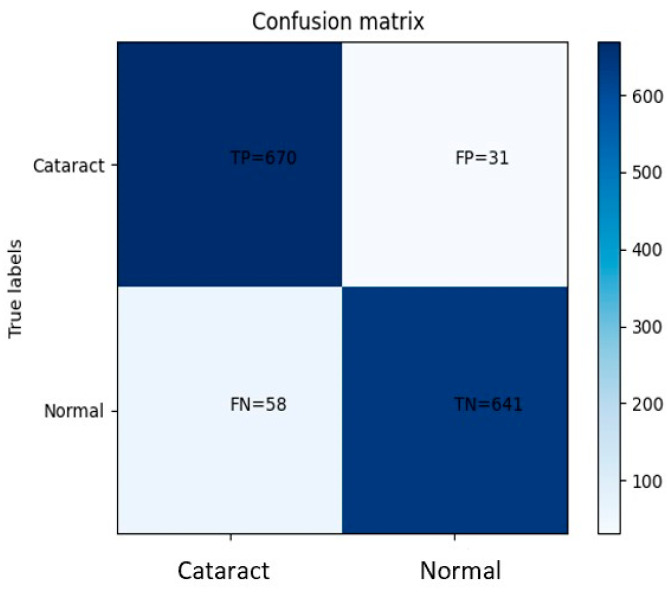
Plot confusion matrix of CNN.

Figure 10 shows that based on the provided confusion matrix, the true positive rate (sensitivity) is calculated as the ratio of correctly identified positive cases (disease) to the total number of actual positive cases. In this case, the sensitivity is determined to be 0.92, indicating a high level of accuracy in correctly identifying eye images with cataract symptoms. Similarly, the true negative rate (specificity) is computed as the ratio of correctly identified negative cases (normal) to the total number of actual negative cases. With a specificity value of 0.95, the model demonstrates a strong ability to accurately classify normal eye images. These results highlight the model’s robust performance in accurately detecting cataract symptoms and distinguishing normal eye images, as evidenced by the high sensitivity and specificity values obtained from the analysis of the confusion matrix.

**Figure 11:**
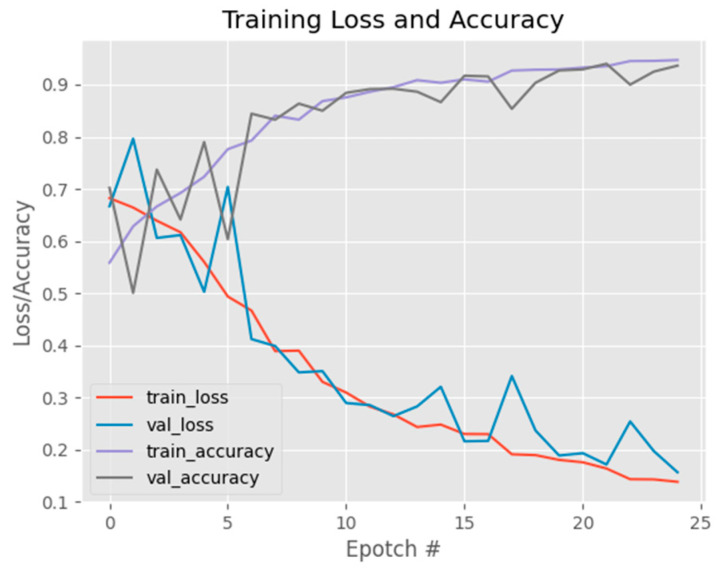
Graph showing accuracy and loss per epoch of the initial model.

Figure 11 shows the initial model exhibited a high accuracy score; however, upon analyzing Figure 10, it became apparent that as the training dataset size increased, the accuracy curve showed continuous improvement, while the validation accuracy curve failed to stabilize. This behavior was similarly observed in the loss curve, where the training loss continued to decrease without the validation loss reaching a steady state. Consequently, these results indicate that the model’s performance is suboptimal. To address this issue, a new parameter configuration was implemented, consisting of a reduced batch size of 16, a fixed number of 25 epochs, a learning rate of 0.1, and the utilization of the binary cross-entropy loss function with the SGD optimizer. With these adjusted parameters, the updated model achieved an accuracy of 0.96 as shown in Table 2. These findings suggest that the modified parameter settings effectively enhanced the model’s performance, resulting in a higher accuracy score. The alterations in batch size, learning rate, loss function, and optimizer played pivotal roles in optimizing the model’s training process, leading to improved classification accuracy in distinguishing between normal and diseased eye images.

Based on the obtained confusion matrix, the classification performance of the model can be evaluated. It correctly classified 676 eye images displaying cataract symptoms while misclassifying 25 images. Additionally, the model accurately identified 662 normal eye images but misclassified 37 images as normal when they actually exhibited cataract symptoms. These results indicate a relatively high accuracy in distinguishing between cataract-affected and normal eyes. However, the presence of misclassifications suggests the need for further improvement. Analyzing the misclassified images can provide valuable insights into the underlying patterns or features that contribute to the incorrect categorization. This analysis can guide the refinement of the model architecture, adjustment of hyperparameters, or augmentation of the dataset to mitigate misclassifications and enhance the overall performance of the model. By iteratively repeating this process, it is possible to achieve a more robust and accurate model for cataract classification.

**Figure 12:**
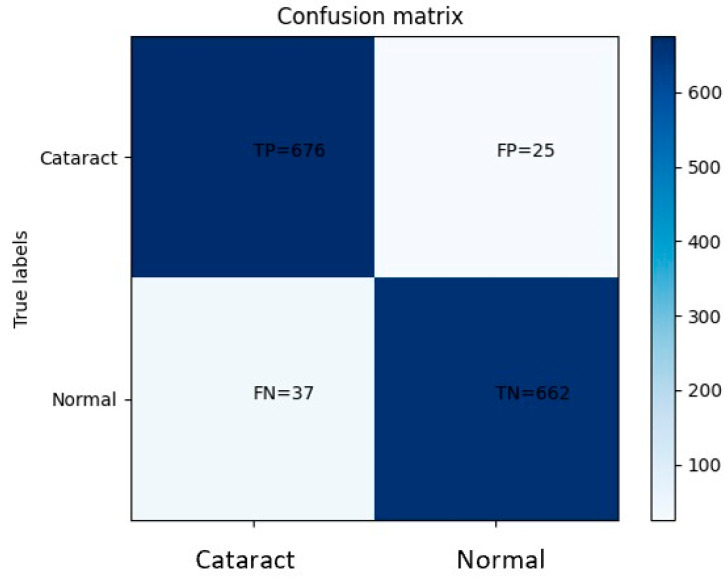
The graph obtained from the plot confusion matrix of CNN.

Figure 12 shows the confusion matrix; the true positive rate, also known as sensitivity or recall, is defined as the proportion of correctly predicted positive instances out of all actual positive instances. In this scenario, the sensitivity is calculated to be 0.95, indicating that the model accurately identified 95% of the diseased cases. Similarly, the true negative rate, also referred to as specificity, measures the proportion of correctly predicted negative instances out of all actual negative instances. The specificity value is determined to be 0.96, indicating that the model correctly classified 96% of the non-diseased cases as negative. These findings highlight the model’s ability to effectively capture the distinctive features associated with cataract symptoms and normal eye images. The high sensitivity and specificity values signify the model’s proficiency in accurately identifying both positive (disease) and negative (non-disease) instances, underscoring its overall performance and reliability in distinguishing between the two categories. 

**Figure 13:**
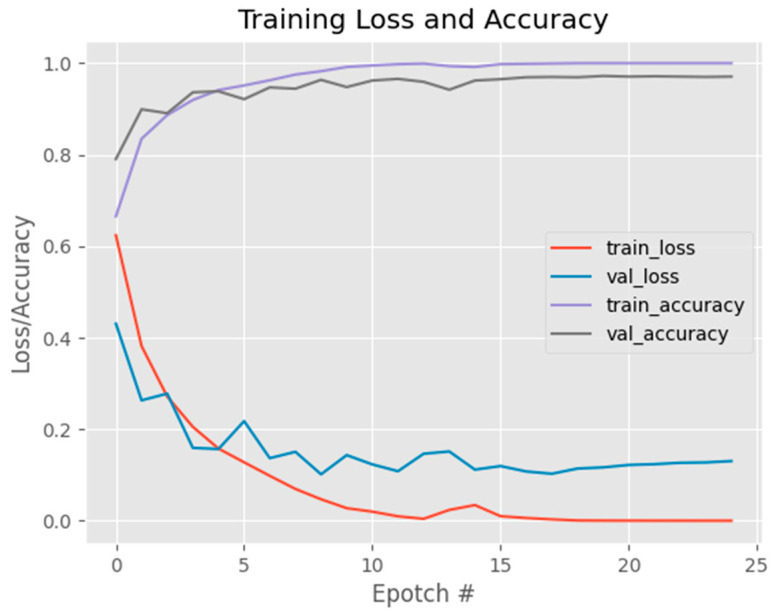
Graph showing accuracy and loss per epoch of the proposed model.

**Figure 14:**
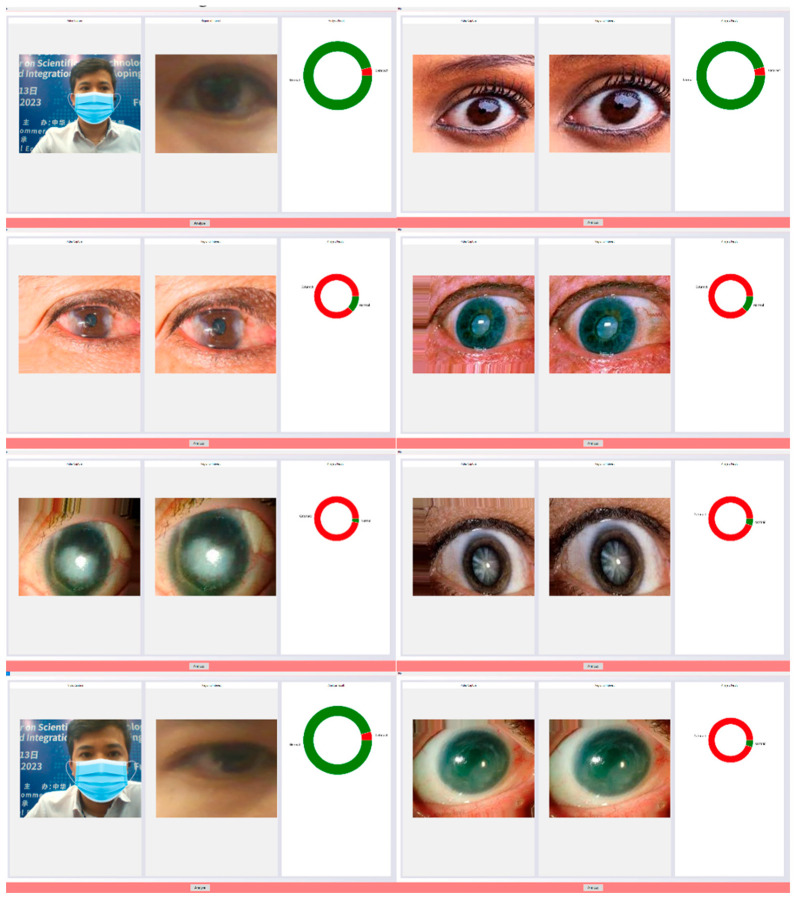
Results of the cataract detection experiment.

Figure 13 shows the findings from Figure 14, revealing an interesting trend in the model’s performance as the training dataset size increases. The accuracy graph demonstrates a positive correlation, showing a consistent improvement in the model’s ability to correctly classify the data. This indicates that the model becomes more accurate with the inclusion of more training instances. Similarly, the loss graph exhibits a negative correlation, indicating a reduction in the model’s error as more data are utilized for training. Notably, the validation accuracy and validation loss graphs demonstrate a more stable behavior compared to the previous parameter settings. This stability suggests that the model’s performance is not overly influenced by the specific training instances and can generalize well to unseen data. Consequently, the model’s configuration appears to be more suitable, as it achieves higher accuracy and lower loss while maintaining a balanced performance on the validation set. Overall, these observations highlight the effectiveness of the current model configuration in capturing the underlying patterns of the data and producing reliable predictions. The increasing accuracy, decreasing loss, and stable validation metrics collectively indicate that the model is well-optimized and capable of making accurate predictions on both training and unseen data.

Figure 14 presents the experimental results of cataract detection, showcasing the graphical user interface utilized in the experiment. The graphical interface serves as the input medium for the image processing system to perform cataract detection. The system promptly returns the detection results to the user through a user-friendly and convenient cataract detection device. Notably, this system exhibits a high level of accuracy in cataract detection, surpassing current state-of-the-art studies.

### 3.3. Comparison of Analysis Results

The empirical findings presented in Table 3 elucidate the conspicuously elevated efficacy of the LeNet-CNN model in relation to other methodologies within the ambit of ocular disease classification. The LeNet-CNN model attains an impressive 96% accuracy rate, markedly outperforming its counterparts such as the SVM at 92%, decision tree at 86%, random forest at 90%, Naive Bayes at 79%, and neural network at 87%. Moreover, scrutinizing the true positive rate (sensitivity) reveals LeNet-CNN’s remarkable attainment of 95%, while SVM registers at 91%, decision tree at 89%, random forest at 91%, Naive Bayes at 77%, and neural network at 87%. Parallelly, the evaluation of the true negative rate (specificity) underscores LeNet-CNN’s substantial achievement at 96%, while SVM reaches 94%, decision tree at 84%, random forest at 89%, Naive Bayes at 81%, and neural network at 86%. These comprehensive metrics collectively underscore the LeNet-CNN model’s discernibly heightened precision and effectiveness in the correct classification of both positive and negative instances. This salient performance renders the LeNet-CNN model the preeminent choice for discerning pathological deviations in ocular disorders. The amalgamation of superior accuracy, augmented sensitivity, and enhanced specificity consolidates its standing as an optimal tool for the meticulous identification and categorization of aberrant ocular conditions.

Based on the comparative analysis presented in Table 4, the research employing image processing technology for corneal abnormality detection exhibits notable clarity and accuracy in its results. The process of segregating corneal abnormalities using the proposed technique demonstrates a higher level of accuracy compared to other existing research endeavors. This can be attributed to the utilization of a more extensive and comprehensive dataset, surpassing the scale employed in previous studies, consequently resulting in improved accuracy levels. Our proposed model outperforms other works regarding the accuracy at 96% except only [60] in which it achieved 96.8% highlighted in bold.

In the context of research focusing on the design and development of software for corneal abnormality detection through image processing technology, the LeNet-CNN model was chosen due to its inherent efficiency and exceptional accuracy. By employing the LeNet-CNN model, the research achieved a heightened level of accuracy, surpassing the benchmarks set by other studies. Therefore, the proposed technique, which includes the incorporation of the LeNet-CNN model, convincingly demonstrates its efficacy and precision in the corneal abnormality detection process. This substantiates the efficiency and accuracy of the proposed technique, accentuating its capability to accurately identify and classify corneal abnormalities within the tested samples. The study’s findings underscore the potential of the proposed technique, particularly with the utilization of the LeNet-CNN model, in significantly enhancing the detection of corneal abnormalities.

## 4. Discussions

In this work, we present an investigation and the development of a system aimed at detecting and classifying corneal abnormalities using advanced image processing technology. The primary objectives of this research endeavor encompass the following key points: (1) understanding the process of corneal abnormality discrimination utilizing image processing techniques; (2) designing, constructing, and advancing a sophisticated software program for detecting corneal abnormalities; and (3) evaluating the performance and efficacy of the developed program in detecting corneal abnormalities. A thorough review and analysis of pertinent theories and previous research works were conducted to assimilate and adapt the relevant knowledge into the design and development processes. A comprehensive dataset comprising 3500 images, including both normal and corneal abnormality cases, was meticulously curated for training and testing purposes. Prior to training the dataset, preprocessing methodologies were applied to optimize the image data, ensuring its suitability for subsequent machine learning procedures. The LeNet model, a type of CNN, was selected as the primary model for training the prepared dataset. The performance and accuracy of the LeNet model were systematically benchmarked against alternative techniques proposed in the literature. The results unequivocally demonstrated the superior accuracy and effectiveness of the LeNet-CNN model over other approaches. Furthermore, the LeNet model was deployed in the design and implementation of an intuitive graphical user interface (GUI) that seamlessly interfaces with the dedicated LeNet database, purposefully constructed for this research initiative. This integrated system facilitates user-friendly interactions and efficient retrieval of corneal abnormality detection results. The culmination of this research underscores the immense potential and efficacy of the proposed methodology in accurately identifying and discerning corneal abnormalities. By leveraging the power of the LeNet-CNN model and the bespoke software program developed, this research significantly advances the state-of-the-art in corneal abnormality detection, thus holding tremendous promise for clinical applications and enhancing diagnostic precision in the field of ophthalmology.

Remarkable findings have emerged based on the comprehensive investigation into the process of discerning anomalies associated with corneal diseases, employing image processing technology coupled with the application of machine learning techniques, specifically the LeNet-CNN model. Notably, the LeNet-CNN model has exhibited superior performance compared to other relevant research endeavors, boasting an accuracy rate of 96%, a sensitivity rate of 95%, and a specificity rate of 96%. This outcome is in alignment with the stipulated research objectives. Furthermore, a detailed exposition elucidating the intricacies of the LeNet-CNN model underscores its multifaceted computational framework, drawing inspiration from the intricate analysis of human neurological data during the transmission of neural signals. This computational paradigm capitalizes on the extraction and integration of sub-features, contributing to a holistic analysis of image data. Consequently, the selection of the LeNet-CNN model for designing a corneal disease anomaly detection methodology utilizing image processing technology serves as an apt choice, with the obtained results attesting to the concordance with the research objectives.

The LeNet-CNN model boasts several salient advantages that render it highly suitable for the discernment of corneal disease anomalies, as follows:

1. Efficacious foundational architecture: The LeNet-CNN model has evolved from seminal investigations and empirical experiments dating back to 1998. Its basic architecture rests on the CNN, a highly refined structure renowned for its computational prowess in image processing. Consequently, leveraging the LeNet-CNN model for corneal disease anomaly discernment engenders a credible and efficient approach;
2. Substantiation of salient sub-features: The LeNet-CNN model adopts an intricate analytical approach to image analysis, heavily influenced by the examination of human data in the context of neural information transmission. By virtue of employing methodologies that enable the identification and aggregation of pertinent sub-features, the LeNet-CNN model possesses the capacity to pinpoint critical and indispensable attributes pertaining to corneal disease anomalies at an elevated level of precision;
3. Discerning class categorization with accuracy: The LeNet-CNN model exhibits remarkable accuracy in the classification of image classes, as evidenced by an analysis of the dataset utilized in the research, with accuracy, sensitivity, and specificity rates reaching 96%, 95%, and 96%, respectively. These compelling values underscore the LeNet-CNN model’s proficiency and efficiency in categorizing corneal disease anomalies;
4. Versatility in diverse research domains: Beyond its utility in the classification of corneal disease anomalies, the LeNet-CNN model exhibits remarkable flexibility, rendering it adaptable for employment in a range of related research endeavors that necessitate robust image processing capabilities.

In conclusion, the LeNet-CNN model demonstrates exceptional accuracy in discerning corneal disease anomalies, complemented by an intricate analytical process adept at identifying critical attributes. Consequently, its implementation in research for the classification and diagnosis of corneal diseases in real-world settings proves highly conducive to the achievement of research objectives. Future research can explore more comprehensive data, including detailed severity levels and protein deposition locations, to advance the system into a multiclassification framework. Additionally, efforts can be directed towards refining the training process and image enhancement techniques by acquiring high-quality, noise-free images. Environmental factors, such as lighting conditions and image positions, impacted the system’s performance, suggesting the potential for a notification mechanism to guide users on optimal image capture.

## Figures and Tables

**Table 1:** Accuracy results of CNNs.

	Precision	Recall	F1 Score	Support	Accuracy
0	0.92	0.96	0.94	701	0.94
1	0.95	0.92	0.94	699	0.94

**Table 2:** Results from the CNN testing.

	Precision	Recall	F1 Score	Support	Accuracy
0	0.95	0.96	0.96	701	0.96
1	0.96	0.95	0.96	699	0.96

**Table 3:** The performance comparison between the LeNet-CNN and other machine learning techniques.

		SVM	Decision Tree	Random Forest	Naive Bayes	Neural Network	LeNet-CNN
Accuracy		0.92	0.86	0.90	0.79	0.87	0.96
Sensitivity		0.91	0.89	0.91	0.77	0.87	0.95
Specificity		0.94	0.84	0.89	0.81	0.86	0.96

**Table 4:** Numerical results of related studies using digital camera images.

References	Method	Accuracy	Training Data	Testing Data	Cross-Validation
[29]	Single Layer Perceptron	85%	30	20	No
[59]	Support Vector Machine	88.39%	125	49	No
[61]	K-nearest Neighbor	94.5%	80	80	No
	Support Vector Machine	69.4%			
[60]	Support Vector Machine	**96.8%**	58	36	No
	Artificial Neural Network	92.3%			
[62]	K-nearest Neighbor	83%	78	42	No
	Support Vector Machine	75.2%			
	Naïve Bayes	76.6%			
[63]	Convolutional Neural Network	78%	100	30	No
**Our model**	LeNet-CNN	**96%**	80	20	No

## Data Availability

The Cataract Image dataset is available at https://www.kaggle.com/alexandramohammed/cataract-image (accessed on 5 January 2023). The Cataract Detection App—Using CNN Neural Network is available at https://github.com/KrishnaRauniyar/CataractDetectionApp-UsingCNN-NeuralNetwork/tree/master/Cataract(accessed on 5 January 2023).
